# BuB: a builder-booster model for link prediction on knowledge graphs

**DOI:** 10.1007/s41109-023-00549-4

**Published:** 2023-05-23

**Authors:** Mohammad Ali Soltanshahi, Babak Teimourpour, Hadi Zare

**Affiliations:** 1 Department of Information Technology, Faculty of Industrial and Systems Engineering, Tarbiat Modares University, Tehran, Iran; 2 Department of Information Technology, University of Tehran, Tehran, Iran

**Keywords:** Link prediction, Knowledge graph completion, BuB, Relationship builder and booster, Discriminative fine-tuning

## Abstract

Link prediction (LP) has many applications in various fields. Much research has been carried out on the LP field, and one of the most critical problems in LP models is handling one-to-many and many-to-many relationships. To the best of our knowledge, there is no research on discriminative fine-tuning (DFT). DFT means having different learning rates for every parts of the model. We introduce the BuB model, which has two parts: relationship Builder and Relationship Booster. Relationship Builder is responsible for building the relationship, and Relationship Booster is responsible for strengthening the relationship. By writing the ranking function in polar coordinates and using the nth root, our proposed method provides solutions for handling one-to-many and many-to-many relationships and increases the optimal solutions space. We try to increase the importance of the Builder part by controlling the learning rate using the DFT concept. The experimental results show that the proposed method outperforms state-of-the-art methods on benchmark datasets.

## Introduction

The massive amount of data available on the internet has attracted many researchers to work on various fields such as computer vision (Giveki et al. 2017; Montazer et al. 2017), transfer learning (Giveki et al. 2022), data science (Mosaddegh et al. 2021; Soltanshahi et al. 2022), social networks (Ahmadi et al. 2020), knowledge graph (Molaei et al. 2020). Knowledge graphs have many applications in fields such as health (Li et al. 2020), finance (Huakui et al. 2020), education (Shi et al. 2020), cyberspace security (Zhang and Liu 2020), social networks (Zou 2020). Some examples of knowledge graphs is google knowledge (Steiner et al. 2012), KG-Microbe (Joachimiak et al. 2021), kg-covid-19 (Reese et al. 2021), Biological Knowledge Graphs (Caufield et al. 2023), OwnThink (https://www.ownthink.com/), Bloomberg knowledge graph (Meij 2019), and Clinical Knowledge Graph (Santos et al. 2020). Knowledge graphs are widely used by the tech giants such as Google, Facebook, Netflix, and Siemens (Rikap et al. 2021).

Therefore, knowledge graphs are used in various fields and industries, and completing the knowledge graph impacts them. LP aims to complete knowledge graphs. Many application methods use LP methods like recommender systems (Zhou et al. 2020).

The knowledge graph is a set of facts. A fact connects two entities by relation and has three components: head, relation, and tail. LP in the knowledge graph helps to complete the knowledge graph and extract new facts from the existing facts. Many LP methods seek to provide an embedding for each fact components and evaluate its plausibility using a ranking function. There are three types of models based on ranking function: (1) Tensor Decomposition Models, (2) Geometric Models, (3) Deep Learning Models (Rossi et al. 2021). Geometric models are less efficient than the two types of models. Deep learning models are more complex in terms of parameters; consequently, model training requires vast amounts of data (Ostapuk et al. 2019). This article focuses on models based on tensor decomposition.

The most popular method among tensor decomposition methods is ComplEx (Lacroix et al. 2018). After the ComplEx method, many methods tried to improve it. These studies focus on the generalization of the ComplEx model (Gao et al. 2021a; Zhang et al. 2020), model mapping in the polar coordinates (Sun et al. 2019), introduce new regularization expression (Zhang et al. 2020) and sampling methods (Zhang et al. 2019). Nevertheless, to the best of the authors' knowledge, no method has directly addressed handling one-to-many and many-to-many relationships, the importance of the parameters, and their learning speed. To this end, we use Transfer learning and the DFT concept and rewrite the ranking function in polar coordinates.

Transfer learning has many applications in various fields, such as natural language processing and image processing (Zhuang et al. 2020). Transfer learning technique uses neural network models that have already been trained on huge databases to solve smaller problems. One of the applications of DFT is in transfer learning (Howard and Ruder 2018). In DFT, different components have different training rates, but we have used one learning rate and controlled the change ratio of the two sets of parameters by applying a coefficient.

We use a proposition: to have a good relationship, one should build it first and then strengthen it. By writing the ranking function in the polar coordinates, we divide the embedding of a fact into two main parts: angle (as builder part), and length (as booster part). A relationship (or a fact) is built when its relation angle equals the difference between its head and tail angles. A relationship (or a fact) is strengthened when the length of its relation, head, and tail increases. Using this concept and the concept of DFT, we propose a method in which the speed of the learning angle is more important than length.

One of the most critical problems in LP methods is handling one-to-many and many-to-many relationships. For example, many people are born in the United States and complete the relationship <?, “born in”, USA>. Our proposed method solves the origin of this problem. On the other hand, this method increases the number of optimal solutions and compresses the space of optimal solutions. The innovations of this article are:

1. Introduce the BuB model and divide the model parameters into the relationship Builder and Booster parts.
2. Write a ranking function in polar coordinates and increase the importance of the relationsh­ip Builder part using the DFT concept.
3. Provide direct solutions for one-to-many and many-to-many relationship handling.
4. Increase predictive performance in low dimensional embedding so that the difference in performance between the embedding dimensions 100 and 2000 is negligible and insignificant.
5. The proposed method has outperformed models based on tensor decomposition.

The remainder of this paper is organized as follows: Sect. “Literature review” reviews LP methods in knowledge graphs and related works. We describe our proposed method in Sect. “BuB model” and evaluate our method on popular KGs in Sect. “Experimental results”, and finally, Sect. “Conclusion and research directions” is devoted to the conclusion and future research directions.

## Literature review

The knowledge graph is a multi-graph *KG* = (E, R, G) where E is the set of entities, R is the set of relations, G is the set of edges in the knowledge graph, and G ⊆ E × R × E. Each edge in the knowledge graph is called a fact that connects an entity (head of relationship or object) to another entity (tail of relationship or subject) through a relation. Each fact is a triad <h, r, t> where h denotes head, r represents relation, and t denotes tail.

Knowledge graph has many applications in different fields (Zou 2020). The main issue in knowledge graphs is information incompleteness, affecting the performance of knowledge graph methods (Arora 2020). It has two solutions: (1) Link Prediction(LP), an essential task to complete the knowledge graphs (Wang et al. 2021). (2) Integrate the knowledge graph with other homogeneous knowledge graphs. It requires knowledge graph alignment, and some newer knowledge graph alignment methods use link prediction (Sun et al. 2018; Wang et al. 2018; Yan et al. 2021; Tang et al. 2020).

The main aim of LP in knowledge graphs is to predict missing and new facts by observing the existing facts and current information. LP in knowledge graphs seeks to complete the fact triple in which a component is unknown. Accordingly, there are three types of link prediction problems:

1. **Predict tail of the fact** $< h,r,? >$ Where the head and relation are known, and the tail is unknown.
2. **Predict relation of fact** $< h,?,t >$ Where the head and tail are known, and the relation is unknown.
3. **Predict head of the fact** $< ?,r,t >$ Where the relation and tail are known, and the head is unknown.

Link prediction methods are divided into two main categories (Meilicke et al. 2019).

1. Embedding-based methods
2. Rule-based methods

This article discusses embedding-based methods, and please refer to Meilicke et al. (2019) for more details about rule-based methods. In embedding-based methods, entities and relations are represented by a vector or matrix. A ranking function estimates the plausibility of a fact (Wang et al. 2021).. Then a loss function is introduced using the ranking function, and the loss function is minimized using machine learning algorithms.

Consider the X set of training facts labeled with L. Loss functions are classified into three categories:

1. **Margin-based loss functions** In these loss functions, training facts have two categories: positive facts and negative facts. The goal is to make a 2λ-margin between the rank of positive facts and the rank of negative facts so that the rank of positive facts is close to λ and the rank of negative facts is close to − λ (Bordes et al. 2013; Wang et al. 2014; Lin et al. 2015; Kazemi and Poole 2018).
2. **Binary Classification loss functions** In this category, the link prediction problem is converted to a binary classification problem, and the binary classification loss functions are used (Vu et al. 2019; Nguyen et al. 2017).
3. **Multi-class Classification loss functions** In this category, the link prediction problem is converted to a multi-class classification problem, and the multi-class classification loss functions are used (Lacroix et al. 2018; Gao et al. 2021a; Dettmers et al. 2018; Balažević et al. 2019).

After learning the loss function, it is time to evaluate the proposed method. Suppose Y is the set of rankings obtained. To evaluate the proposed method, we use Hits@k or H@k, MRR metrics, defined as follows (Rossi et al. 2021).

**H@k** Ratio of facts whose rank is equal to or less than k.

1 $$\begin{array}{*{20}c} {H@k = \frac{{\left| {\left\{ {x{|}x \in Y \;and\; x < k} \right\}} \right|}}{\left| Y \right|} } \\ \end{array}$$

**MRR** Average of the inverse of the obtained ranks.

2 $$\begin{array}{*{20}c} {{\text{MRR}} = \frac{1}{\left| Y \right|}\mathop \sum \limits_{y \in Y} \frac{1}{y}} \\ \end{array}$$

Three class of embedding-based method exists:

1. Geometric methods
2. Tensor Decomposition methods
3. Deep Learning methods

Tensor Decomposition methods are simple, expressive, and fast and have higher predictive performance than geometric methods (Rossi et al. 2021). Deep Learning methods are more complex and lower predictive results than tensor decomposition methods. So, tensor decomposition methods are more practical than deep learning methods.

### Geometric methods

**TransE** The first LP method is TransE (Bordes et al. 2013), which is one of the geometric methods. This model defines the ranking function as $f\left( {h,r,t} \right) = -\parallel h + r - t \parallel$, and its geometric interpretation is translation. Geometric translate means the fact $\left\langle {h,r,t} \right\rangle$ exists when from h gets to t, with the vector r. The TransE method cannot handle one-to-many, many-to-one, and many-to-many relationships.

**TransR and TransH** After TransE, methods such as TransR (Lin et al. 2015) and TransH (Wang et al. 2014) were introduced. TransH maps h and t to a hyperplane. TransR maps h and t to a hyperplane that is a function of r.

**RotatE** The RotatE method (Sun et al. 2019) uses the rotation concept to define the ranking function as $f\left( {h,r,t} \right) = -\parallel h \odot r - t\parallel$ which $h,r,t \in {\mathbb{C}}^{d}$ and the size of each element of the vector r is one. The authors (Sun et al. 2019) also introduce the pRotatE method with ranking function $f\left( {h,r,t} \right) = - {\text{sin}}\left( {h + r - t} \right)$.

### Tensor decomposition methods

**Distmult** In 2014, the first tensor decomposition method, DistMult, was proposed (Yang et al. 2014). In the DistMult method, the components $h,r,t \in {\mathbb{R}}^{d}$, and the ranking function is:

$$f\left( {h,r,t} \right) = \left( {h \otimes r} \right) . t$$

where $\otimes$ denotes the multiplication of corresponding elements, and “.” denotes the inner product.

**ComplEx** The ComplEx method (Trouillon et al. 2016) map DistMult into complex space. The ranking function of ComplEx is $f\left( {h,r,t} \right) = \left( {h \otimes r} \right) .\overline{t}$, where $h,r,t \in {\mathbb{C}}^{d}$ and $\overline{t}$ is the complex conjugate of t.

In 2018, the ComplEx-N3 method (Lacroix et al. 2018), proposed a new regularization term N3 for the ComplEx method to improve it. Inspired by the ComplEx method, researchers propose many models such as SimplE (Kazemi & Poole, 2018), AutoSF (Y. Zhang, et al., 2020), QuatE (L. Gao, et al., 2021) and QuatDE (H Gao, et al., 2021).

**SimplE** In the SimplE method (Kazemi and Poole 2018), each entity is represented by two vectors, one for when the entity is the head of a fact and one for when the entity is the tail of a fact. So each relation is represented by two vectors, one for relations in the regular direction and one for the reverse direction. This method is fully expressive but could not increase the efficiency of link prediction compared to the ComplEx method.

**AutoSF** In the AutoSF method (Zhang et al. 2020), the authors introduced a new algorithm to find a specific configuration for each KGs. They use Low-dimensional embedding with short training to find the best configuration. Nevertheless, it cannot be used in large datasets such as Yago3-10 because training with low-dimensional embedding on large datasets is highly time-consuming.

**QuatE and QuatDE** In QuatE (Gao et al. 2021a), the authors map it into quaternion space (one value with three imaginary values) to generalize the ComplEx model. In QuatDE (Gao et al. 2021b), the authors use a dynamic mapping strategy to separate different semantic information and improve the QuatE method.

**Tucker** Tucker method (Balažević et al. 2019) is a powerful and linear method based on tensor decomposition. Tucker, like the SimplE method, is fully expressive. Several methods, such as ComplEx, RESCALE, DistMult, and SimplE, are all specific types of Tucker. In this method, a three-dimensional tensor, ${\mathcal{W}} \in {\mathbb{R}}^{d \times d \times d}$, encodes information on the knowledge graph. The ranking function is defined as follows:

3 $$\begin{array}{*{20}c} {f_{r} \left( {h,t} \right) = W \times_{1} h \times_{2} r \times_{3} t} \\ \end{array}$$

where $\times_{n}$ is the tensor multiplication in the nth dimension, the ${\mathcal{W}}$ W is like memory and holds all the information of the knowledge graph. It is the essential component of the Tucker method and makes it powerful. ${\mathcal{W}}$ requires a lot of memory and limits d so that d cannot be more than 200.

### Deep learning methods

Deep learning has many applications in many areas, including link prediction (Razzak et al. 2018; Miotto et al. 2018; Chalapathy and Chawla 2019; Zhang et al. 2018). Deep learning models have strong representations and generalization capabilities(Dai et al. 2020).

**ConvE** For the first time (Dettmers et al. 2018) used the convolution network for the link prediction task. This method uses a matrix to represent entities and relations. First, it concatenates head and relation, feeds the resulting matrix to a 2D convolution layer, and operates 3 × 3 filters to create different feature mappings. It feeds feature mappings to a dense layer for classification. This method achieves good results in WN18 and FB15k databases by providing an inverse model for inverse relationships.

**ConvKB** The ConvKB method (Nguyen et al. 2017) seeks to capture global relations and the translational characteristics between entities and relations. In this method, each entity and relation are a vector, and a 3-column matrix represents each fact. Like the ConvE method, it inputs the result matrix to a convolution layer and applies the 1-by-3 matrix to generate feature mappings. It feeds feature mappings to a dense layer for classification.

**ConvR** ConvR method (Jiang et al. 2019) uses filters specific to each relationship instead of public filters in the convolution layer. Each entity is a two-dimensional matrix, and each relation is a convolution layer filter. It feeds the entity matrix to the convolution layer and applies the relation-specific filters to it to produce the feature mapping. It feeds feature mappings to a dense layer for classification.

**CapsE** In CapsE method (Vu et al. 2019), similar to the ConvKB, each fact is a 3-column matrix. This matrix enters to convolution layer, and 1 × 3 filters are applied to it to produce feature mapping. Then the feature mapping is fed to a capsule layer and converted into a continuous vector for classification.

## BuB model

The proposed model has two parts: relationship builder and relationship booster. Relationship Builder tries to build the relationship, and Relationship Booster tries to strengthen the relationship. By writing the ranking function in the polar coordinates, we define our ranking function as follows:

4 $$\begin{array}{*{20}c} {f\left( {h,r,t} \right) = \underbrace {{R_{h} \odot R_{r} \odot R_{t} }}_{{{\text{booster}}}}.\underbrace {{{\text{cos}}\left( {\theta_{h} + \theta_{r} - \theta_{t} } \right)}}_{{{\text{builder}}}}} \\ \end{array}$$

where $\left( {R_{h} , \theta_{h} } \right)$, $\left( {R_{r} , \theta_{r} } \right)$ and $\left( {R_{t} , \theta_{t} } \right)$ represent h, r, and t in the polar coordinates. We call the first part of the ranking function a relationship booster and the second part a relationship builder.

The expression $\theta_{h} + \theta_{r} - \theta_{t}$ is very similar to the ranking function of the TransE method. The TransE method is powerful to handle one-to-one relationships but cannot handle one-to-many, many-to-one, and many-to-many relationships. To overcome this problem, we introduce the following ranking function $f^{n}$

5 $$\begin{array}{*{20}c} {f^{n} \left( {h,r,t} \right) = R_{h} \odot R_{r} \odot R_{t} .\cos \left( {n\left( {\theta_{h} + \theta_{r} - \theta_{t} } \right)} \right)} \\ \end{array}$$

where n is the root factor or frequency of fact. If n = 1, the ranking function is equal to the ranking function of the ComplEx method written in polar coordinates.

Relationships in childhood are different from relationships in adulthood. For example, marriage and teaching relationships in a university do not belong to childhood. On the other hand, different entities have different relationships. Politicians and actors have different relationships. The authors believe each entity and relation have different frequencies, and a relationship is established between two entities at the right frequency. Therefore, to describe an entity with a different life cycle or different social role, it is recommended that we represent it at different frequencies and learn related embeddings.

### Lemma

Consider a knowledge graph $KG = \left( {E, R, G} \right)$ that has been trained with the ranking function $f^{n}$ and the embedding of size 2d, and the suboptimal embedding $E^{*}$ and $R^{*}$ has been obtained. The number of embeddings with the same result as $E^{*}$ and $R^{*}$ is greater than $n^{{d\left( {\left| E \right| + \left| R \right|} \right)}}$.

### Proof

The set $E_{i}^{*}$ and $R_{j}^{*}$ that $i < n^{{d\left( {\left| E \right|} \right)}}$ and $j < n^{{d\left( {\left| R \right|} \right)}}$ are defined as follows.

$$\begin{aligned} & E_{i}^{*} = \left\{ {\left( {R_{k} , \theta_{k}^{^{\prime}} } \right){|}\left( {R_{k} , \theta_{k} } \right) \in E^{*} , 1 \le k \le \left| E \right|, \theta_{k}^{^{\prime}} = \theta_{k} + \frac{{2\left( i \right)_{d}^{k} \pi }}{n}} \right\} \\ & R_{j}^{*} = \left\{ {\left( {R_{k} , \theta_{k}^{^{\prime}} } \right){|}\left( {R_{k} , \theta_{k} } \right) \in R^{*} , 1 \le k \le \left| R \right|, \theta_{k}^{^{\prime}} = \theta_{k} + \frac{{2\left( j \right)_{d}^{k} \pi }}{n}} \right\} \\ \end{aligned}$$

where $\left( . \right)_{d}^{k}$ is the k-th digit of the given number in base d. Obviously $f^{n} \left( {R_{k} , \theta_{k}^{^{\prime}} } \right) = f^{n} \left( {R_{k} , \theta_{k} } \right)$. Therefore, the results obtained for $E_{i}^{*}$ and $R_{j}^{*}$ are the same as the results $E^{*}$ and $R^{*}$, and the number of pairs $E_{i}^{*}$ and $R_{j}^{*}$ is equal to $n^{{d\left( {\left| E \right| + \left| R \right|} \right)}}$.

Increasing the value of n increases the number of sub optimal answers and therefore it is expected that the rate of convergence of the method increases. Large n (more than 30) provides circumstances for overfitting.

On the other hand, $n > 1$ helps the builder part to grow faster than the booster part. Let’s consider following equations:

6 $$\begin{array}{*{20}c} {\frac{{\partial f^{n} }}{{\partial \theta_{{h_{i} }} }} = - nR_{{h_{i} }} R_{{r_{i} }} R_{{t_{i} }} sin\left( {n\left( {\theta_{{h_{i} }} + \theta_{{r_{i} }} - \theta_{{t_{i} }} } \right)} \right) } \\ \end{array}$$

7 $$\begin{array}{*{20}c} {\frac{{\partial f^{n} }}{{\partial h_{{h_{i} }} }} = R_{{r_{i} }} R_{{t_{i} }} cos\left( {n\left( {\theta_{{h_{i} }} + \theta_{{r_{i} }} - \theta_{{t_{i} }} } \right)} \right) } \\ \end{array}$$

where $h_{i}$ denotes the ith element of h, $r_{i}$ is ith element of r, and $t_{i}$ is ith element of t. The above equation shows that $f^{n}$ increases speed of the learning θ, n times.

8 $$\begin{array}{*{20}c} {\frac{{\frac{{\partial f^{n} }}{{\partial \theta_{{h_{i} }} }}}}{{\frac{{\partial f^{n} }}{{\partial R_{{h_{i} }} }}}} = - nR_{{h_{i} }} \frac{{sin\left( {n\left( {\theta_{{h_{i} }} + \theta_{{r_{i} }} - \theta_{{t_{i} }} } \right)} \right)}}{{cos\left( {n\left( {\theta_{{h_{i} }} + \theta_{{r_{i} }} - \theta_{{t_{i} }} } \right)} \right)}} } \\ \end{array}$$

In an equal conditions when $tan\left( {n\left( {\theta_{h} + \theta_{r} - \theta_{t} } \right)} \right) = 1$ and $R_{{h_{i} }} \ll 1$, then changing $\theta_{{h_{i} }}$ is much less than changing $R_{{h_{i} }}$. In other words, changing angles do not affect the output $f^{n}$. To solve this problem, we extend $f^{n}$ ranking function as follows,

9 $$\begin{array}{*{20}c} {f_{g}^{n} \left( {h.r.t} \right) = g(R_{h} ) \odot g\left( {R_{r} } \right) \odot g(R_{t} ).cos\left( {n\left( {\theta_{h} + \theta_{r} - \theta_{t} } \right)} \right)} \\ \end{array}$$

where g is a derivative function. Therefore,

10 $$\begin{array}{*{20}c} {\frac{{\frac{{\partial f_{g}^{n} }}{{\partial \theta_{{h_{i} }} }}}}{{\frac{{\partial f_{g}^{n} }}{{\partial R_{{h_{i} }} }}}} = - n\frac{{g\left( {R_{{h_{i} }} } \right)}}{{g^{\prime}\left( {R_{{h_{i} }} } \right)}}\frac{{sin\left( {n\left( {\theta_{{h_{i} }} + \theta_{{r_{i} }} - \theta_{{t_{i} }} } \right)} \right)}}{{cos\left( {n\left( {\theta_{{h_{i} }} + \theta_{{r_{i} }} - \theta_{{t_{i} }} } \right)} \right)}}} \\ \end{array}$$

For given function $g$, If the ratio $n\frac{{g\left( {R_{{h_{i} }} } \right)}}{{g^{\prime}\left( {R_{{h_{i} }} } \right)}}$ is greater than one, then the effect of the angle on function f will be greater than the length. If $g\left( x \right) = {\text{e}}^{nx}$, the ratio will be equal to one, and angle and length has the same effect on f.

The experimental results have shown that the use of the introduced functions $g$ has no significant effect on predictive performance of our method. In this article, we used only $f^{n}$ to demonstrate the power of the method.

## Experimental results

We use an i7 processor, ram 32, and rx2080-ti graphics and perform tests on five popular KGs:

1. **WN18 (Bordes et al. **2013**)** It contains 40,943 entities, 18 relations, and 141,442 facts and is extracted from the WordNet dataset.
2. **FB15k (Bordes et al. **2013**)** It contains 14,951 entities, 1345 relations, and 483,142 facts and is built from the Freebase database.
3. **FB15k-237 (Toutanova and Chen 2015)** It is a subset of the FB15k dataset and contains 14,541 entities, 237 relations, and 272,115 facts. The authors selected 401 relations with the most facts and then deleted those equivalent or inverse.
4. **WN18RR (Dettmers et al. **2018**)** It is a subset of the WN18 dataset and contains 40,943 entities, 11 relations, and 86,835 facts. The authors remove inverse or similar relations from WN18 to create WN18RR.
5. **YAGO3-10 (Dettmers et al. **2018**)** It is a subset of the YAGO3 dataset and contains 123,182 entities, 37 relations, and 1,079,040 facts. Only entities from the Yago3 dataset with at least ten relations have been selected to create this collection.

We use three well-known metrics H@1, H@10, and MRR to evaluate the proposed method. See Rossi et al. (2021) for more detailed information about these metrics. The hyperparameters setting is the same as the ComplEx-N3 method.

It is necessary to answer the following questions to justify the proposed method:

Q1. How to choose the root factor?

Q2. What is the performance of the proposed method in low-dimensional embedding?

Q3. What is the performance of the proposed method compared to state-of-the-art methods?

In the following subsections, we answer the above questions.

### Q1. How to choose root factor?

**Fig. 1:**
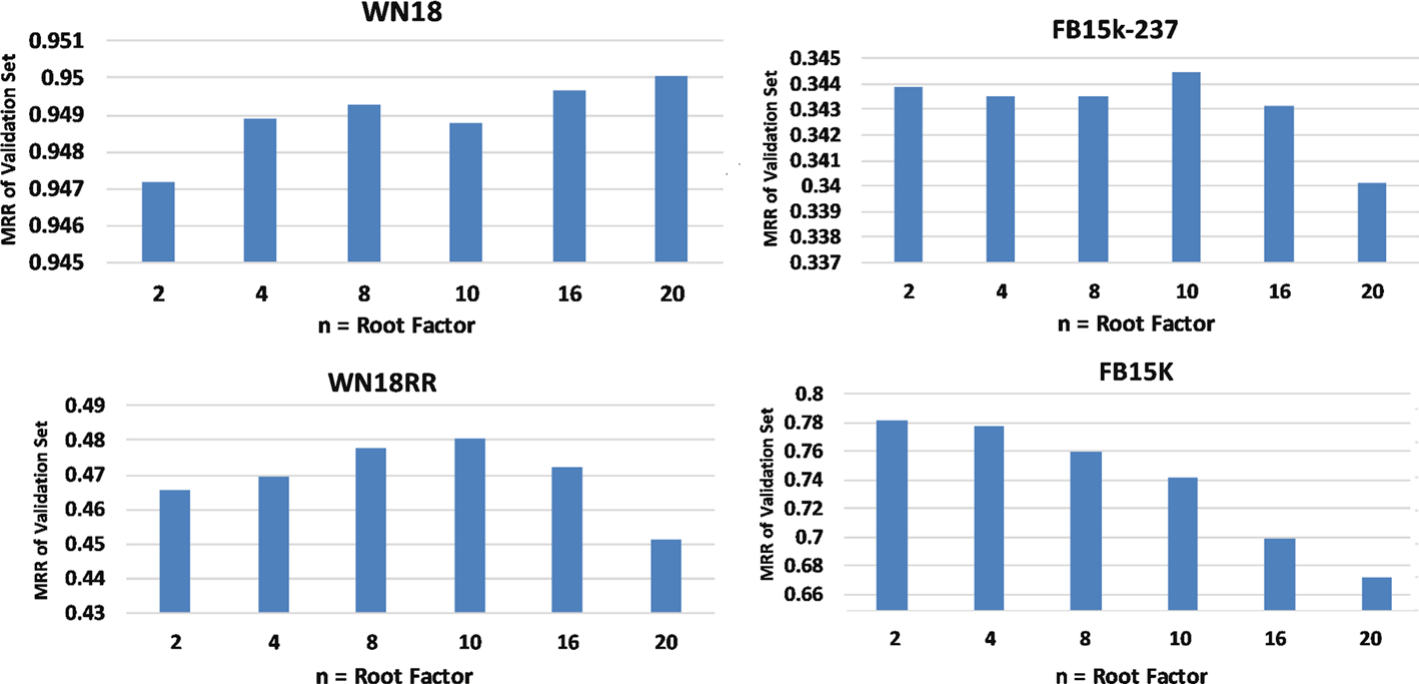
MRR results of the BuB method with embedding length d = 50 and maximum epoch = 100

Experiments on datasets show that using a root factor greater than two increases performance. However, the performance decreases by increasing the value of n from somewhere. Figure 1 represents the BuB method's results on different datasets with d = 50 and different values of n. In this experiment, values of n are 2, 4, 8, 10, 16, and 20, all divisors of 360.

In the WN dataset, by increasing the value of n, the MRR value increases, and the best value for n is 20. In FB15k-237 and WN18RR datasets, the best value is n = 10, and the best value is 2 in the FB15k dataset.

### Q2. What is the performance of the proposed method in low-dimensional embedding?

Since the ComplEx method is one of the best tensor decomposition methods and the proposed method for n = 1 is similar to the ComplEx method, we compare our method with ComplEx method.

**Fig. 2:**
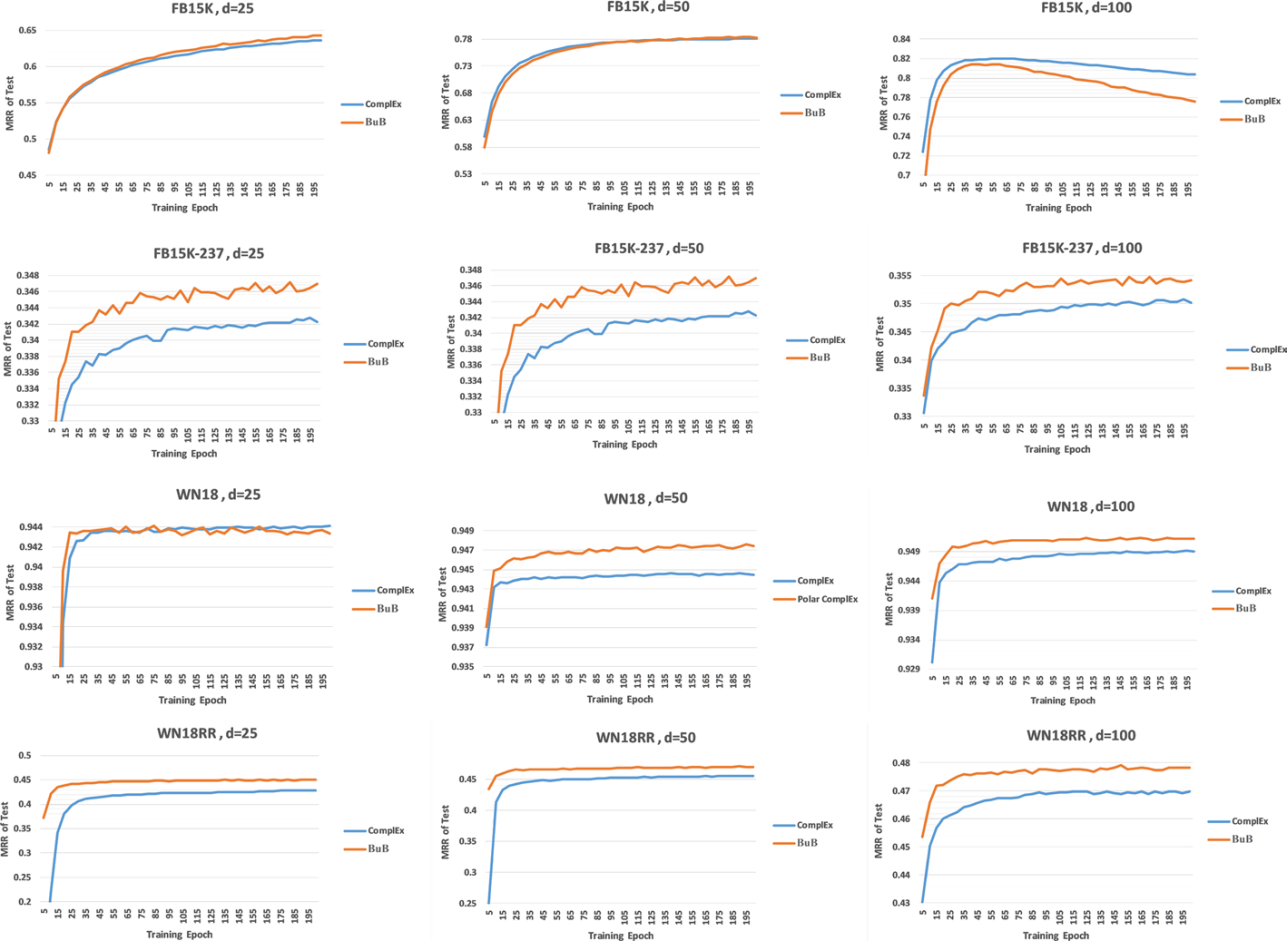
Comparison of the ComplEx method and the BuB with embedding 25,50 and 100 and n = 10

Figure 2 shows that BuB attained better results than ComplEx in low-dimensional embedding. In FB15k, d = 100 diagram, both methods have overfitted after epoch 55, and the overfitting speed in the BuB is more than the original method. FB15k results show that n is not well selected and should be reduced, and as mentioned, the best n for the FB15k dataset is 2.

**Fig. 3:**
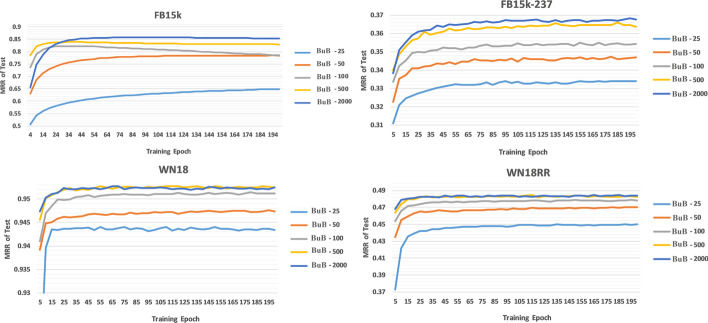
MRR results on different datasets

Figure 3 shows that the results in embedding dimension 100 are so good and are comparable to the embedding dimension 2000.

### Q3. What is the performance of the proposed method compared to state-of-the-art methods?

Table 1 shows that the BuB outperforms state-of-the-art methods in all datasets. The best method is shown in boldface and the second one is shown with underline. The main competitors of the proposed method are AutoSF and QuatDE. For ComplEx-N3, SimplE, AnyBURL, TuckER, RotatE, ConvE, ConvR, ConvKB, and CapsE methods, we use results in the review article (Rossi, et al., 2021), and we use corresponding articles for the QuatE, QuatDE and AutoSF methods.

**Table 1:** Comparison of the BuB with state-of-the-art methods

	FB15k			WN18			FB15k-237			WN18RR			YAGO3-10		
	H@1	H@10	MRR	H@1	H@10	MRR	H@1	H@10	MRR	H@1	H@10	MRR	H@1	H@10	MRR
ComplEx-N3	81.56	90.53	0.848	94.53	95.5	0.949	25.72	52.97	0.349	42.55	52.12	0.458	50.48	70.35	0.576
SimplE	66.13	83.63	0.726	93.25	94.58	0.938	10.03	34.35	0.179	38.27	42.65	0.398	35.76	63.16	0.453
TuckER	72.89	88.88	0.788	94.64	95.8	0.951	25.9	53.61	0.352	42.95	51.4	0.459	46.56	68.09	0.544
RotatE	73.93	88.1	0.791	94.3	96.02	0.949	23.83	53.06	0.336	42.6	57.35	0.475	40.52	67.07	0.498
AutoSF	82.1	91	0.853	94.7	96.1	0.952	26.7	55.2	0.36	**45.1**	56.7	0.49	50.1	**71.5**	0.571
QuatE	71.1	90	0.782	94.5	95.9	0.95	24.8	55	0.348	43.8	58.2	0.488	–	–	–
QuatDE	–	–	–	94.4	96.1	0.95	26.8	**56.3**	0.365	43.8	**58.6**	0.489	–	–	–
ConvE	59.46	84.94	0.688	93.89	95.68	0.945	21.90	47.62	0.305	38.99	50.75	0.427	39.93	65.75	2429
ConvKB	11.44	40.83	0.211	52.89	94.89	0.709	13.98	41.46	0.230	5.63	52.50	0.249	32.16	60.47	1683
ConvR	70.57	88.55	0.773	94.56	95.85	0.950	25.56	52.63	0.346	43.73	52.68	0.467	44.62	67.33	2582
CapsE	1.93	21.78	0.087	84.55	95.08	0.890	7.34	35.60	0.160	33.69	55.98	0.415	0.00	0.00	60,676
AnyBURL	81.09	87.86	0.835	94.63	95.96	0.951	24.03	48.93	0.324	44.93	55.97	0.485	45.83	66.07	0.528
BuB	**82.5**	**91**	**0.856**	**94.8**	**96.5**	**0.9534**	**27.2**	0.558	**0.367**	**45.1**	58.25	**0.496**	**51.35**	71.45	**0.584**

## Conclusion and research directions

We introduce relationship builder and relationship booster expressions in the ranking function and use the DFT concept to increase the speed of relationship builder expressions.

A weakness of our method is the settings of the root factor, which the authors showed that the best n could be obtained by experimenting with low dimensional embedding, but it cannot be used in large datasets. The BuB is simple, has good performance in low dimensional embedding, and outperforms state-of-the-art methods in the WN18, WN18RR, FB15k-237, FB15k, and YAGO3-10 datasets.

The following suggestions are for future research:

- Research on $f_{g}^{n}$ functions to achieve higher performance.
- Provide an adaptive method to adjust the n parameter during training.
- Generalized ranking function as $F_{g}^{n} = \mathop \sum \nolimits_{k = 1}^{n} w_{k} f_{g}^{k}$

## Data Availability

Datasets used for this study are public and included in Lacroix et al. (2018).
